# Autophagy in HCV Infection: Keeping Fat and Inflammation at Bay

**DOI:** 10.1155/2014/265353

**Published:** 2014-08-05

**Authors:** Tiziana Vescovo, Giulia Refolo, Alessandra Romagnoli, Fabiola Ciccosanti, Marco Corazzari, Tonino Alonzi, Gian Maria Fimia

**Affiliations:** ^1^National Institute for Infectious Diseases “Lazzaro Spallanzani” IRCCS, IRCCS, 00149 Rome, Italy; ^2^Department of Biology, “Tor Vergata” University of Rome, 00173 Rome, Italy; ^3^Department of Biological and Environmental Sciences and Technologies (DiSTeBA), University of Salento, 73100 Lecce, Italy

## Abstract

Hepatitis C virus (HCV) infection is one of the main causes of chronic liver disease. Viral persistence and pathogenesis rely mainly on the ability of HCV to deregulate specific host processes, including lipid metabolism and innate immunity. Recently, autophagy has emerged as a cellular pathway, playing a role in several aspects of HCV infection. This review summarizes current knowledge on the molecular mechanisms that link the HCV life cycle with autophagy machinery. In particular, we discuss the role of HCV/autophagy interaction in dysregulating inflammation and lipid homeostasis and its potential for translational applications in the treatment of HCV-infected patients.

## 1. Introduction

Hepatitis C virus (HCV) infection is a major global health problem. Almost 200 million people are infected worldwide, with 3/4 million new infections and 350,000 deaths per year [1, 2]. HCV has a marked ability to establish chronic infection in the liver. Acute infection is often asymptomatic, with spontaneous immune-mediated clearance occurring in only 15%–30% of individuals [3]. Over a time span of years, chronic infection causes a series of liver damages, that is, steatosis, inflammation, fibrosis, and cirrhosis, which significantly increase the risk of developing hepatocellular carcinoma [4].

The HCV world is facing a revolution in terms of therapeutic approaches. First generation direct-acting antivirals (DDAs) are now available in clinics and a large series of new DDAs and host-targeted agents are in advanced clinical trials, with the expectation of curing more than 90% of infections [5]. However, understanding the mechanisms by which chronic HCV infection interferes with host metabolic and immune systems will provide important information to identify new host-targeted antiviral strategies for treating patients who are resistant to DDAs, as well as monitoring the evolution of morbidities in long-term infected HCV patients upon viral clearance.

HCV does not cause a direct cytopathic effect on host cells; most of the related liver dysfunctions are considered a consequence of the virus's ability to dysregulate host processes [6]. In particular, to establish chronic infection, HCV mainly interferes with two important cellular processes, lipid metabolism [7, 8] and immune response [9], by not fully characterized molecular mechanisms. Recent evidence has indicated direct involvement of the autophagy pathway in mediating both positive and negative effects on these processes [10–12]. Here, we will summarize recent advances in understanding the role of autophagy in HCV infection and discuss their translational potential for developing novel therapeutic strategies in order to potentiate viral clearance and prevent HCV-related diseases.

## 2. HCV Life Cycle

HCV is a blood-borne virus of the Flaviviridae family that replicates primarily in hepatocytes. The HCV genome is composed of a positive, single-stranded RNA of approximately 9.6 Kb, containing a long, open reading frame (ORF) flanked by nontranslated regions (NTRs) at 5′ and 3′ ends that regulate RNA translation and replication [13]. HCV RNA encodes a single polyprotein precursor of about 3,000 amino acids, which is cotranslationally and posttranslationally processed by cellular and viral proteases. Polyprotein cleavage products include the structural proteins (the nucleocapsid protein core and the envelope glycoproteins E1 and E2) and nonstructural proteins (p7, NS2, NS3, NS4A, NS4B, NS5A, and NS5B) involved in various processes which are essential for completion of the HCV life cycle [14].

HCV entry in hepatocytes is mediated by the interaction of the viral envelope proteins with several cell surface receptors and entry cofactors including glycosaminoglycans, LDL receptor, tetraspanin CD81, scavenger receptor class B member I, tight junction proteins claudin1 and occludin, and the cholesterol uptake receptor Niemann-Pick C1-like 1 [15]. HCV particles are then internalized by clathrin-mediated endocytosis [16] and viral and endosomal membrane fuse, in a pH-dependent manner, to release viral nucleocapsid into the cytoplasm where the uncoating occurs [17].

HCV RNA is delivered to endoplasmic reticulum, where viral translation is mediated by an internal ribosomal entry site located in the 5′ NTR. Among nonstructural proteins, NS4B and NS5A are able to induce ER membrane alteration, called membranous web, that provide a scaffold for the assembly of the HCV replication complex and protection from host immune defenses [18, 19]. HCV replication is catalyzed by the viral RNA-dependent-RNA polymerase NS5B that allows for the synthesis of new positive strand RNAs, in cooperation with the other nonstructural proteins, through the formation of negative strand replicative intermediates [20]. The formation of new virus assembly is then triggered by the relocalization of core protein and NS5A from ER membranes to cytoplasmic lipid droplets where viral particles are assembled. Maturation and release of HCV particles are closely linked to the very low density lipoprotein (VLDL) synthesis and secretion pathways [21]. Indeed nascent virions associate with pre-VLDL particles to form lipoviroparticles (LVPs) containing apolipoproteins (ApoB, apoE, apoC, and apoA-I), [22, 23]. LVPs then pass through the Golgi and are released from the cells by the secretory pathway [13].

## 3. Autophagy and HCV Infection

The term autophagy refers to a series of processes that allow for the degradation of intracellular components by delivering them into lysosomes [24]. In most cases, double-membrane vesicles, termed autophagosomes, engulf the target material and fuse with lysosomes (macroautophagy) [25]. Alternatively, substrates are directly entrapped in endosomal/lysosomal membrane invaginations (microautophagy) [26, 27] or internalized upon binding to lysosomal receptors (chaperone-mediated autophagy) [28]. Each step of the autophagy process is regulated by specific genes (ATG genes), originally identified in yeast and mostly conserved in higher eukaryotes [29]. The Ulk1 kinase is the first protein in the autophagy cascade into which both positive and negative pathways converge, such as those mediated by the AMPK and mTOR kinases, respectively [30, 31]. Ulk1 activates the Beclin 1/Vps34 complex, which generates PI3P-rich membrane domains allowing for the nucleation of autophagosome precursor structures, known as phagophores [32–34]. Subsequently, two ubiquitin-like conjugation systems mediate the recruitment of ATG12-ATG5 and LC3 proteins to the phagophore allowing for its expansion and closure to form the mature autophagosome [24]. Finally, autophagosomes fuse to endocytic vesicles to form multivesicular bodies and eventually to lysosomes, where their content is degraded by a variety of hydrolases and monomers released for recycling [35].

Although autophagy was originally described as a nonspecific process induced in response to nutrient deprivation, it is now evident that many selective forms have evolved to recognize and degrade damaged, supernumerary, or unwanted substrates. Consistently, signaling pathways triggering selective autophagy as well as substrate receptors specific for unique targets have been identified [36]. For example, removal of damaged mitochondria is activated by the recruitment of Pink1 and Parkin proteins on the outer membrane [37, 38]. Moreover, ubiquitinated protein aggregates are recognized by adaptor proteins SLR (sequestosome 1/p62-like receptors) that share the ability to bridge ubiquitin chains to the autophagosome protein LC3 [39, 40].

The emerging complexity in the field of selective autophagy provides new hints for understanding how autophagy alterations could be associated to the development of a wide range of human diseases [41–43]. In this regard, an important example is represented by the multifaceted role played by autophagy in infectious diseases. Indeed, autophagy plays a crucial role both in the innate and adaptive immunity [44]. On one hand, it acts as a defense mechanism by directly recognizing and degrading pathogens [11]. On the other hand, autophagy participates in the activation of intracellular and systemic immune responses, primarily by making pathogen antigens accessible to immune receptors [11]. Conversely, pathogens have developed many strategies to usurp autophagy to favor persistent infection [45, 46].

HCV is highly successful in establishing chronic infection. Since 2008, several studies have been conducted to clarify the contribution of autophagy alterations in establishing chronic HCV infection and in the onset of associated diseases [47–49]. In particular, two aspects of the autophagy process have been extensively investigated in the context of HCV infection: (1) whether autophagy is induced or inhibited by HCV, (2) whether basal or induced autophagy plays a role in HCV infection.

The emerging scenario, not devoid of conflicting results, indicates the presence of a delicate balance between proviral and prohost functions of autophagy depending on the activation of selective types of this process.

## 4. Is Autophagy Induced or Inhibited in HCV Infected Cells?

**Figure 1:**
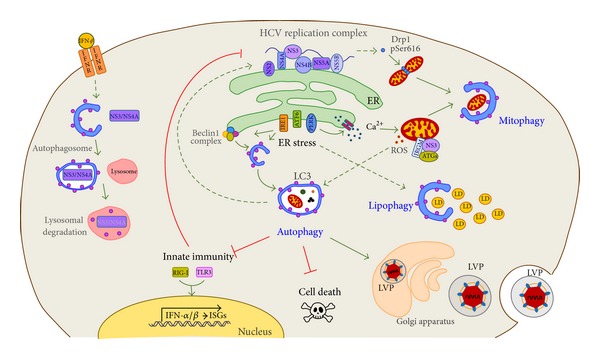
A schematic drawing showing the interplay between HCV and autophagy. HCV nonstructural (NS) proteins assemble with the viral genome on the endoplasmic reticulum (ER) membranes to form the replication complex. Viral replication alters ER homeostasis leading to ER stress, which, in turn, activates the unfolded protein response (UPR) through ATF6, IRE1, and PERK proteins. UPR plays a major role in the induction of autophagy, either directly or indirectly by affecting cellular redox balance. In fact, ER stress causes calcium release from ER that results in impaired mitochondrial activity coupled to excessive production of reactive oxygen species (ROS). Damaged mitochondria are thus targeted for degradation via mitophagy. Mitophagy is also stimulated by HCV by activating the mitochondrial fission protein Drp1. Notably, the mitochondrial protein IRGM interacts with both viral and autophagy proteins (ATGs) and is required for autophagy induction. HCV exploits the autophagic process to accomplish different steps of its life cycle such as translation, replication, assembly, and release of lipoviroparticles (LVP). These effects are, at least in part, indirect since autophagy represses the intracellular innate immune pathways, thus inhibiting IFN production. On the other hand, autophagy could also act as defense mechanism against HCV, since when induced by IFN-*β* causes the degradation of the viral protein NS3/NS4A. Importantly, autophagy is crucial for preventing pathogenesis induced by HCV. Indeed, a type of autophagy selective for lipids (lipophagy) protects cells from an excessive lipid accumulation triggered by HCV (LD: lipid droplets). Moreover, infected cells are more prone to death when autophagy is inhibited. Mitophagy is selectively involved in the control of HCV infection, innate immunity, and cell death (not shown in the figure, see text for details).

The presence of an increased number of autophagosomes in HCV-infected cells has been reported by several groups, based on observations in either cell culture infection systems or liver samples from patients with chronic HCV infection [50–55]. However, whether HCV induces a functional or incomplete autophagy process remains controversial. Initial works reported an accumulation of autophagosomes in the absence of increased turnover of long-lived proteins and degradation of the autophagic cargo p62 [51]. Conversely, using similar* in vitro* infection or replication systems, proper fusion between autophagosomes and lysosomes has been reported, as well as the occurrence of LC3 protein degradation within the lysosome, indicating that a functional autophagy flux occurs in the presence of HCV [54]. A possible explanation for these contradictory results came from the observations that degradation of specific autophagic targets is increased in HCV-infected cells, such as damaged mitochondria and lipid deposits, suggesting that HCV induces selective rather than bulk autophagy [56, 57] (Figure 1). Moreover, it has recently been proposed that the ability of HCV to inhibit the autophagic flux may depend on the viral genotype. In fact, HCV replicon cells carrying a genotype 1b strain Con1 exhibit an incomplete acidification of autolysosomes, while those with the genotype 2a strain JFH1 have no alterations [58].

## 5. How Does HCV Modulate Autophagy?

Multiple interactions between HCV and autophagy proteins have been identified. By yeast two-hybrid assays, NS5B, NS5A, and p7 were found to interact with ATG5, ATG12, and FIP200, respectively [59, 60]. Moreover, immunoprecipitation assays revealed the ability of NS4B to copurify with the Beclin 1-associated lipid kinase Vps34, as well as p7 with Beclin 1 [61, 62]. Interestingly, in most cases these interactions result in autophagy induction [60, 63].

Many evidences point to the fact that autophagy is indirectly induced by cellular stress activated by HCV infection. In particular, the modulation of autophagy by HCV has been primarily linked to the activation of endoplasmic reticulum (ER) stress (Figure 1). HCV infection induces chronic ER stress in hepatocytes, which has been functionally linked to presence of clinical dysfunctions observed in patients, including steatosis, cell death, and immune escape [64]. ER stress is known to activate a signaling network, called the unfolded protein response (UPR), to cope with the damaging agents. UPR consists of three distinct pathways, identified by their main regulatory factors, namely, the activating transcription factor 6 (ATF6), the inositol-requiring enzyme 1 (IRE1), and the double-stranded RNA-activated protein kinase-like ER kinase (PERK) [65] (Figure 1). HCV-induced ER stress activates all three UPR pathways and the inhibition of each of them was shown to decrease autophagy levels in infected cells [51, 54, 66], although conflicting results have been reported on the contribution of IRE1 to UPR-induced autophagy [67] How HCV, ER stress, and autophagy are functionally linked has begun to be clarified. The core protein has been shown to activate both ATF6 and PERK pathways that, in turn, increase the expression of ATG12 and LC3 genes [63]. HCV is also able to transcriptionally upregulate Beclin 1 expression [68]; however, whether this is dependent on UPR or not has not been investigated. Moreover, the role of Beclin 1 complex remains unclear with reports showing that HCV-induced autophagy is either dependent or independent of its activity [52, 53, 69, 70].

The activation of signaling pathways downstream to ER stress may play a role in the induction of autophagy by HCV. For example, it has been proposed that the induction of autophagy by HCV depends on the inhibition of the AKT/mTOR pathway by UPR [71]. However, discordant results have been reported on the status of mTOR activity in HCV-infected cells [68, 71–73].

An important consequence of ER stress induced by HCV is the alteration of ER calcium homeostasis (Figure 1). Release of calcium from ER to cytosol is known to impair mitochondrial activity, resulting in the accumulation of mitochondrial reactive oxygen species (ROS), which are potent autophagy inducers [74, 75]. In line with this model, induction of mitophagy, a selective form of autophagy for removal of damaged mitochondria, has been observed in HCV-infected cells [56] (Figure 1). Moreover, modulation of the cellular redox state by catalase expression is able to reduce autophagy levels in HCV replicon cells [76]. Interestingly, full-length HCV replicon cells show a decreased antioxidant response when compared to those expressing only the nonstructural proteins, suggesting that autophagy is induced by ROS as a compensative mechanism to the inhibition of host antioxidant pathways by structural proteins [76]. Recently, it has been reported that HCV is able to directly promote mitophagy by inducing mitochondrial fission via phosphorylation of the dynamin-related protein 1(Drp1) [77].

A central role of mitochondria in the induction of autophagy has been also confirmed by a recent study aimed at characterizing common mechanisms of autophagy modulation by different RNA viruses [60, 78]. The authors identify the immunity-associated GTPase family M (IRGM) protein as a potential hub to convey autophagy and viral proteins. IRGM is a mitochondria-associated factor playing a crucial role in the regulation of autophagy in a variety of infections [79, 80]. By two-hybrid screening, IRGM was found to directly interact with NS3/4A and several autophagy proteins, that is, ATG5, ATG10, LC3, and BIF1, and the downregulation of IRGM expression reduces autophagy levels in HCV-infected cells NS5B [60]. Interestingly, also BNIP3, an important regulator of mitochondrial selective autophagy, is able to interact with different HCV proteins, that is, NS2, NS4A, NS5A, and NS5B [60].

It is therefore likely that a combination of different pathways, not all yet characterized, may induce autophagy at different stages of HCV infection. HCV infection activates different pattern recognition receptors [81], which are able to trigger autophagy [11]; however, their contribution in the induction of autophagy during HCV infection remains poorly characterized. Another important yet unexplored aspect is whether HCV could control autophagy by modulating the activity of the cytosolic RNA-sensing protein kinase PKR, which has been reported to regulate virus- and starvation-induced autophagy [82–85]. Intriguingly, HCV is either able to activate (via RNA IRES and core protein) or to inhibit PKR (via NS5A and E2 proteins) at different steps of the viral life cycle [82], which could possibly account for a dual regulation of autophagy activity in the course of infection.

In this regard, an integrated analysis of how autophagy is modulated during different steps of infection will provide useful information to elucidate how HCV intersects the autophagy pathway.

## 6. Is Autophagy Required for HCV Infection?

Several reports have highlighted a strict interdependence between the execution of the HCV life cycle and autophagy activity or the expression of autophagy proteins. It is recognized that inhibition of the autophagy machinery affects the production of new infective particles [53, 69]. However, at which steps autophagy is required for the HCV life cycle remains controversial.

HCV replication was shown to occur either normally or drastically inhibited in autophagy-deficient cells [52, 53, 58, 59, 69]. Discrepancies were also reported with regard to the presence of HCV replication complexes on autophagosomal membranes. Confocal microscopy and immunoelectron microscopy studies showed that HCV NS4B, NS5A, and NS5B proteins and nascent HCV RNA colocalize with autophagy markers and/or autophagosomal structures [59, 70, 86]. In contrast, several other studies failed to detect any significant colocalization [51, 53, 67].

The highly heterogeneous nature of Huh7 cells and derivative clones and the different strategies of HCV expression (RNA transfection versus infection) may account, at least in part for the observed discrepancies. In addition, the interaction between HCV and the autophagy machinery may be regulated in a temporally regulated manner, as suggested by a recent study showing that the colocalization of ATG5 and NS5B is detected at 2 days postinfection, while it is absent at 5 days [59]. This hypothesis is substantiated by data from The Chisari Lab, underlining the involvement of the autophagy machinery in the translation and/or delivery of incoming viral RNA to the translation apparatus, while it becomes dispensable for HCV RNA progeny once replication is established [52]. The authors proposed that the autophagy pathway may provide an initial membranous support for translation of incoming RNA, before accumulation of viral proteins and establishment of the replication complex within the ER-associated membranous web. At these early stages of infection, basal rather than stress-induced autophagy is likely to contribute to HCV infection, since autophagy increase was reported to occur at a relatively late time, when replication has been established [71]. Experimental evidences support the idea that late induction of autophagy, as well as mitophagy, is required to sustain the survival of infected cells, a crucial aspect for a virus able to establish a chronic infection [69, 77].

## 7. Autophagy and the Innate Immune Response in HCV-Infected Cells

HCV has evolved multiple mechanisms to evade innate immunity. In particular, HCV inhibits the interferon (IFN) response, acting both upstream and downstream to IFN production [87]. For example, the viral protease NS3/4A inhibits the retinoic acid-inducible gene I (RIG-I) and toll-like receptor 3 (TLR3) antiviral pathways by proteolytically cleaving the MAVS and TRIF factors [88–91]. Moreover, different HCV proteins are able to interfere with the JAK-STAT pathway activated by IFNs [92–94].

A turning point in elucidating the relationship between autophagy and HCV infection is represented by three recent works demonstrating the involvement of autophagy in repressing the anti-HCV innate immune response (Figure 1). Indeed, the impairment of autophagy, either by targeting ATG genes or the upstream ER stress process, leads to a significant upregulation of the innate immune response to HCV [54, 69, 95]. The suppression of innate immunity during HCV infection relies on the catabolic activity of autophagy, since it was also prevented by interfering with lysosome function [54]. This is different from what reported in cells infected with vesicular stomatitis virus, where the inhibition of IFN-*β* production by autophagy was dependent exclusively on the interaction between the Atg5-Atg12 complex and the RIG-I and IFN-*β* promoter stimulator 1 (IPS-1) proteins [96].

Although the relationship between autophagy and innate immunity in HCV infected cells has been well established, it remains unclear whether the exacerbation of innate immune response is the main cause of the inhibition of HCV infection in autophagy-defective cells. To answer this question, the impact of autophagy inhibition on HCV infection should be tested in cells defective for the major viral sensing pathways.

ER stress and autophagy are also involved in the inhibition of the antiviral response downstream to IFN production by downregulating the expression of type I IFN (IFN-*α*) receptors but not type II (IFN-*γ*) or type III (IFN-*λ*) [95]. In the same context, the expression of the nucleoside transporters ENT1 and CNT1, which are involved in the transport of the antiviral drug Ribavirin (RBV), is also decreased, thus suggesting a possible contribution of autophagy in the partial resistance to type I IFN/RBV-based therapy* in vivo* [95]. On the other hand, it should be underlined that the IFN signaling pathway is preactivated in nonresponsive patients [97, 98], which leads to the hypothesis that interfering with the crosstalk between autophagy and innate immunity may actually represent an advantage for HCV persistence.

Recent data highlighted a further level of complexity in the crosstalk between the interferon pathway and autophagy in the context of HCV infection. By means of transgenic mice expressing HCV NS3/NS4A protein in the liver, it was shown that IFN-*β* is able to trigger autophagy-mediated degradation of the viral protein [99] (Figure 1). Degradation is dependent on the mitochondria-associated antiviral signaling protein MAVS and is specific for IFN-*β*, since it is not observed when mice are treated with IFN-*α*. If confirmed in the context of HCV infection, these results represent an important example of how autophagy may play both positive and negative roles in the innate immune response to HCV infection. In relation to these results, it has been reported that HCV clearance can be induced by treating infected cells with AICAR, an AMP analog capable of activating AMPK, a protein kinase with pleiotropic functions including direct stimulation of the autophagy process [100]. However, the contribution of autophagy in HCV clearance mediated by AMPK remains to be assessed.

HCV infection is not only impaired by the general inhibition of the autophagy process but also by interfering with the induction of mitophagy, a selective type of autophagy activated by the PINK1/Parkin pathway [56, 77].

In light of the autophagy-innate immunity relation, it would be important to test whether mitophagy is also able to hamper the innate immune response. Interestingly, aberrant stimulation of mitochondrial fission by HCV, which leads to mitophagy, results in the repression of interferon response [77]. Alternatively, the inhibition of HCV infection could be a consequence of a dysregulated production of ROS by unremoved damaged mitochondria, which may directly affect the HCV replication machinery or indirectly, the host metabolic pathways required for viral life cycle.

## 8. Autophagy and the Alteration of Lipid Metabolism during HCV Infection

The HCV life cycle is tightly coupled to the lipid metabolism of host cells [7, 19]. HCV virions are bound to lipoproteins, called “lipoviroparticles,” wherein the circulating virus hides and mediates its entry into the hepatocytes through lipoprotein receptors [13]. Moreover, ER-associated lipid droplets are the viral assembly sites where the nucleocapsid protein core and the replication complex interact to initiate capsid assembly [101]. NS5A also interacts with apolipoproteins A and E that are required for both HCV replication and the release of viral particles via VLDL secretion [102–104].

HCV not only associates with intracellular lipid compartments but also induces profound alteration in lipid metabolism leading to steatosis [105]. The core and NS5A expression results in the accumulation of lipid droplets (LD), and polymorphisms in the LD-binding domain of Core correlate with the different steatogenic properties of HCV strains, which are more accentuated in the HCV genotype 3 [106–108]. Core and NS5A also inhibit the microsomal triglyceride transfer protein, a key protein in VLDL assembly, and increase lipogenesis through the activation of sterol regulatory element-binding protein 1, peroxisome proliferator-activated receptor, and retinoid X receptor [8, 109, 110].

Lipophagy has been recently described as a selective type of autophagy dedicated to the degradation of intracellular lipid stores [111, 112]. The contribution of defective autophagy in the onset of lipid dysmetabolic diseases, such as steatosis and atherosclerosis, has recently emerged [10]. Notably, when we analyzed the levels of autophagy in the liver of HCV-infected patients with respect to clinical symptoms, we found an inverse correlation with the presence of microsteatosis [57]. The role of autophagy in the catabolism of lipid stores during HCV infection was confirmed by* in vitro* studies using both HCV-replicon and HCV-infected cells. In these systems, we found that a large fraction of autophagosomes selectively colocalize with lipid deposits, whose levels are increased by viral replication [57] (Figure 1). Moreover, inhibition of autophagy by pharmacological and genetic approaches leads to a substantial increase of intracellular cholesterol deposits, while preventing cholesterol synthesis by statins significantly decreases autophagy levels as well as viral replication [57]. Taken together, these data suggest that inhibition of lipid selective autophagy may contribute to the onset of steatosis in chronically HCV-infected patients.

Many aspects of the role of lipid-selective autophagy in HCV-infected cells require further investigation. For example, it will be interesting to test whether the interaction of core protein with lipid droplets, in particular from HCV genotype 3 with a higher steatogenic potential, is able to interfere with the access of these structures to the autophagic machinery. Moreover, being cholesterol essential for HCV virion assembly and release [8], it is conceivable that lipophagy inhibition may account for the observed defects in HCV infectivity in autophagy-deficient cells by decreasing the availability of recycled cholesterol for the synthesis of novel lipoproteins.

## 9. Perspectives

The dual role played by autophagy in inflammation and lipid metabolism in HCV-infected liver cells raises an important question regarding the contribution of autophagy defects in disease progression towards steatohepatitis, fibrosis, cirrhosis, and hepatocarcinoma in patients with chronic HCV infection. Recent studies not focused on HCV infection show that the onset of steatohepatitis in nonalcoholic fatty liver patients is associated to lower levels of “bulk” autophagy and, in particular, to the accumulation of the autophagy cargo protein p62 [113–115]. In agreement with this observation, hepatocyte-specific atg7 knockout mice show liver damage associated with increases in hepatic TGs and cholesterol [111, 116]. Notably, in these mice, accumulation of p62 accelerates liver damage, which leads to the development of hepatic cancer via persistent activation of the Nrf2 pathway [117–119].

Autophagy has been proposed as a potential target for tackling HCV infection. However, the emerging scenario indicates that many types of selective autophagies are playing a role in the liver homeostasis of both healthy and HCV-infected individuals. Full elucidation of the molecular basis of this selectivity is therefore needed to provide a framework for the rational design of feasible anti-HCV drugs or intervening applications.
